# High-Resolution Conformational Analysis of RGDechi-Derived Peptides Based on a Combination of NMR Spectroscopy and MD Simulations

**DOI:** 10.3390/ijms231911039

**Published:** 2022-09-20

**Authors:** Clementina Acconcia, Antonella Paladino, Maria della Valle, Biancamaria Farina, Annarita Del Gatto, Sonia Di Gaetano, Domenica Capasso, Maria Teresa Gentile, Gaetano Malgieri, Carla Isernia, Michele Saviano, Roberto Fattorusso, Laura Zaccaro, Luigi Russo

**Affiliations:** 1 Department of Environmental, Biological and Pharmaceutical Science and Technology, University of Campania—Luigi Vanvitelli, Via Vivaldi 43, 81100 Caserta, Italy; 2 Institute of Biostructures and Bioimaging, CNR, Via Castellino 101, 80131 Naples, Italy; 3 Advanced Accelerator Applications, A Novartis Company, Via Ribes, 510010 Colleretto Giacosa, Italy; 4 Interdepartmental Center of Bioactive Peptide, University of Naples Federico II, Via Mezzocannone 16, 80134 Naples, Italy; 5 Center for Life Sciences and Technologies (CESTEV), University of Naples Federico II, Via De Amicis 95, 80145 Naples, Italy; 6 Institute of Crystallography, CNR, URT Caserta, Via Vivaldi 43, 81100 Caserta, Italy

**Keywords:** integrin, structure–activity relationship, peptide dynamics, natural-abundance NMR, recognition mechanism

## Abstract

The crucial role of integrin in pathological processes such as tumor progression and metastasis formation has inspired intense efforts to design novel pharmaceutical agents modulating integrin functions in order to provide new tools for potential therapies. In the past decade, we have investigated the biological proprieties of the chimeric peptide RGDechi, containing a cyclic RGD motif linked to an echistatin C-terminal fragment, able to specifically recognize αvβ3 without cross reacting with αvβ5 and αIIbβ3 integrin. Additionally, we have demonstrated using two RGDechi-derived peptides, called RGDechi1-14 and ψRGDechi, that chemical modifications introduced in the C-terminal part of the peptide alter or abolish the binding to the αvβ3 integrin. Here, to shed light on the structural and dynamical determinants involved in the integrin recognition mechanism, we investigate the effects of the chemical modifications by exploring the conformational space sampled by RGDechi1-14 and ψRGDechi using an integrated natural-abundance NMR/MD approach. Our data demonstrate that the flexibility of the RGD-containing cycle is driven by the echistatin C-terminal region of the RGDechi peptide through a coupling mechanism between the N- and C-terminal regions.

## 1. Introduction

Peptides play a crucial role in the regulation of many physiological processes, performing their functions by interacting with specific receptors on the cell [1]. Often, the conformational ensemble sampled by a peptide in solution plays an important role in the modulation of the recognition mechanism by which the peptide interacts with a specific partner [2]. Therefore, comprehension of the biological functions of peptides or proteins is of critical importance to understand the structural peculiarities of the biologically conformational ensemble. Many peptides/proteins perform crucial functions by means of structural heterogeneity. Conformational disorder, under physiological conditions, offers several functional advantages: the possibility to interact with different partners, specific but low-affinity binding, and fine modulation after post-translational modifications [3,4,5]. For such unfolded or partially unfolded peptides, proper conformational analysis requires not only descriptions of average local and global features, but details on the various conformers that contribute to the conformational ensemble and their relative populations [6,7].

**Figure 1:**
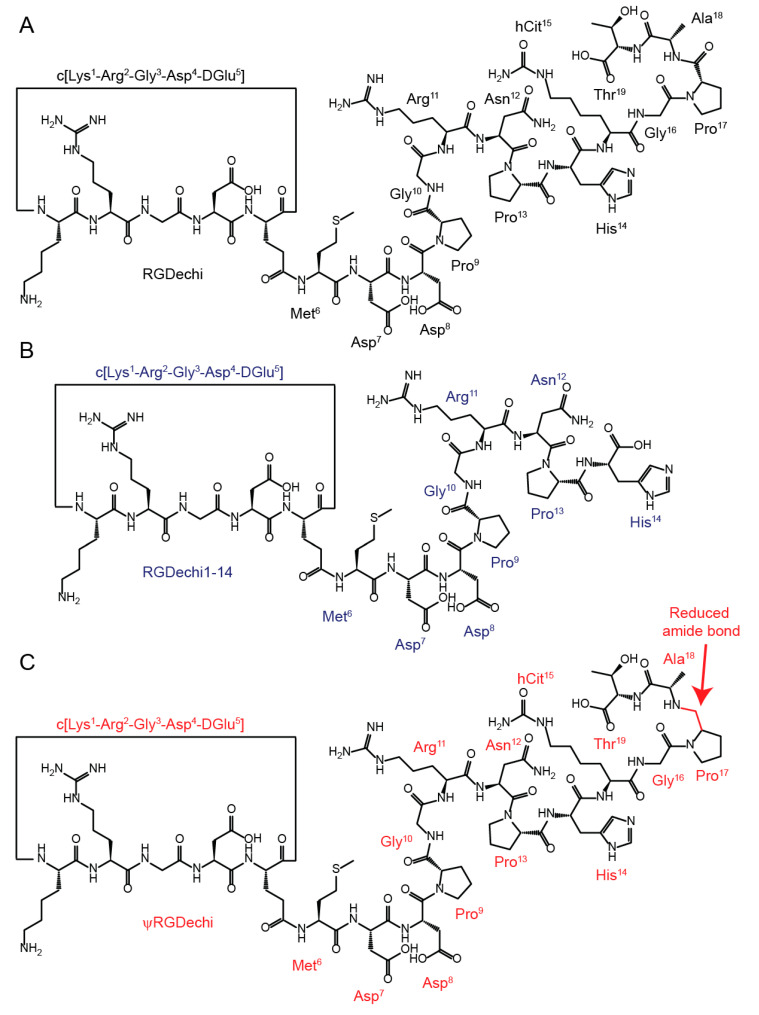
Chemical structure of RGDechi (**A**) and its two derivatives RGDechi1-14 (**B**) and ψRGDechi (**C**).

Therefore, in the case of fully or partially flexible peptides, an accurate structural description is much more challenging, due to their multiplicity of conformational states and dynamic nature. In this framework, to provide a rigorous description of the conformational space sampled by less ordered or flexible peptides in solution, we employed an integrated approach combining structural and dynamical information obtained by natural-abundance nuclear magnetic resonance (NMR) methodologies with molecular dynamics (MD) simulations data. NMR spectroscopy is a powerful tool for investigating the structure and dynamics of peptides and proteins in a solution and their interactions with other molecules [8,9,10,11,12,13]. To perform an accurate high-resolution NMR-based structural and dynamical characterization of peptides, a complete 1H, 15N, and 13C chemical shifts assignment is a fundamental prerequisite [14]. This aspect is particularly important for peptides lacking secondary structure elements, that in solution exist as a highly dynamic heterogeneous ensemble of conformers. Uniform isotope labelling of peptides with 13C and 15N, coupled with double- and triple-resonance NMR experiments allows to derive an in-depth characterization of the structure and dynamic properties of folded and, above all, unfolded peptides [15]. Unfortunately, 15N and/or 13C isotope labelling of peptides containing modified amino acids or cyclic portions is not trivial; therefore, cyclic or unnaturally modified peptides are usually unlabeled. In these cases, natural-abundance NMR techniques represent a unique way to investigate, at atomic level, ensemble-averaged structural properties of peptides in solution [16]. In this scenario, molecular dynamics simulations are valuable tools, complementary to natural-abundance NMR experimental data, that allow an exhaustive description of dynamical processes of peptides at the picoseconds to milliseconds time scale [8,17,18]. We applied our strategy to explore the ensemble-averaged conformational features of two peptides derived by RGDechi (Figure 1A), called RGDechi1-14 (Figure 1B) and ψRGDechi (Figure 1C), respectively. RGDechi is a bifunctional flexible peptide containing a cyclic RGD penta-peptide for integrin binding that is covalently linked by a spacer to a C-term echistatin fragment to confer a high selectivity for the β_3_ integrin subunit [19]. Integrins are a family of heterodimeric membrane receptors, composed of non-covalently associated α and β subunits [20], that are crucial to fundamental cellular functions such as: signaling, cell–cell and cell–extracellular matrix adhesion, and viability. In tumor cells, integrin expression contributes to initiation/progression of cancer and metastasis [21] by accelerating tumor cell migration, invasion, proliferation, and survival [22,23]. Among members of the integrin family, the RGD-binding receptors α_v_β_3_, α_v_β_5_, and α_5_β_1_ play a prominent role in angiogenesis and metastatic dissemination [24]. In vitro and in vivo data [16,25,26,27,28,29,30,31,32] have demonstrated the selective interaction of RGDechi with α_v_β_3_ integrin and structural analysis, based on a combination of NMR methodologies and computational data, highlighted the molecular details of the integrin binding [29]. In particular, the three-dimensional (3D) structural model of the RGDechi/α_v_β_3_ complex, obtained using experimental and computational data, has demonstrated that the recognition mechanism of α_v_β_3_ by RGDechi is mainly modulated by the residues located within the RGD cycle and it is further stabilized by the region encompassing hCit15-Thr19, playing a crucial role in RGDechi selectivity for the α_v_β_3_ [29]. These findings were supported by adhesion assay data obtained using a truncated version of RGDechi (RGDechi1-14) (Figure 1B), indicating that the removal of the last five C-terminal amino acids abolishes the α_v_β_3_ binding selectivity [25]. Yet, NMR structural and dynamical investigation of the conformational ensemble explored through RGDechi in its free form suggested that the recognition of α_v_β_3_ integrin by the peptide may occur by means of a conformational selection mechanism driving the binding of the RGD cycle to the integrin, followed by an induced fit model by which the binding conformation of the acyclic C-terminal region is assumed upon interaction [16]. Recently, to improve the potential of RGDechi as a bioprobe platform in melanoma tumors, we have designed an RGDechi derivative peptide called ψRGDechi (Figure 1C), in which a reduced amide bond ψ[CH2-NH] was introduced. Interestingly, this chemical modification improved protease stability, preserving the binding selectivity for α_v_β_3_ integrin [33]. Here, to understand how to increase the integrin binding selectivity of RGDechi-derived peptides by chemical modifications, we performed a high-resolution description of the conformational space sampled by RGDechi1-14 and ψRGDechi using an integrated natural-abundance NMR/MD approach.

## 2. Results and Discussion

### 2.1. Chemical Shifts Assignment of RGDechi1-14 and ψRGDechi

**Figure 2:**
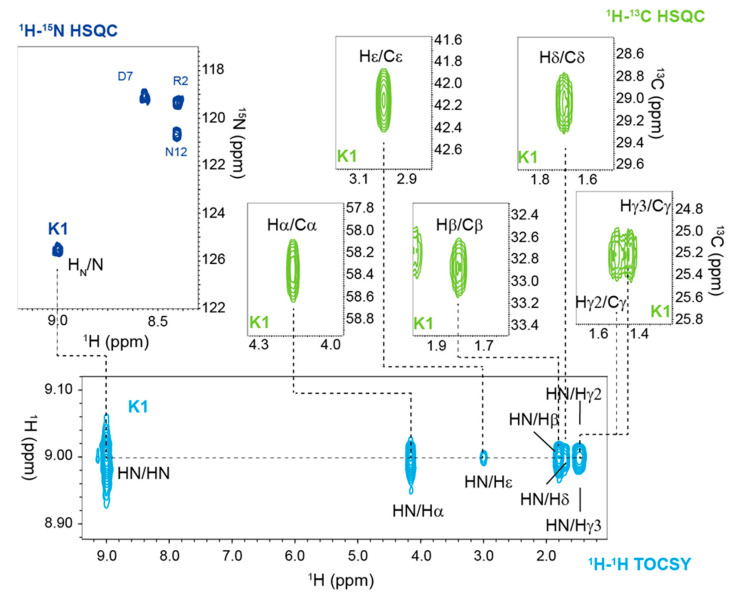
Chemical shifts assignment strategy. Illustration of the procedure used to assign 1H, 13C, and 15N chemical shifts for RGDechi1-14 and ψRGDechi by exploiting the natural isotopic abundance. For example, 2D heteronuclear (1H-15N HSQC and 1H-13C CT HSQC) and homonuclear 1H-1H TOCSY NMR spectra analyzed to assign Lys1 resonances of RGDechi1-14. Data acquisition was performed for RGDechi1-14 and ψRGDechi peptides at 298 K on 600 MHz spectrometer.

A nearly complete assignment of 1H, 13C, and 15N chemical shifts at 298 K was obtained for both peptides (Tables S1–S4) using a previously reported strategy [16] in which homonuclear and heteronuclear 2D NMR spectra were simultaneously analyzed. In particular, the identification of the spin systems and the assignment of 1H resonances were carried out by combining the homonuclear 2D [1H-1H] TOCSY and 2D [1H-1H] ROESY experiments (Figure S1A–D), whereas 15N and 13C chemical shift assignments were obtained by exploiting the natural isotopic abundance through the optimization of the 2D 1H-15N HSQC and 1H-13C CT HSQC pulse sequences. These two latter experiments were analyzed, as illustrated in Figure 2, using the proton chemical shifts obtained from the inspection of the 2D homonuclear spectra.

### 2.2. Chemical Shifts Analysis of RGDechi1-14 and ψRGDechi

**Figure 3:**
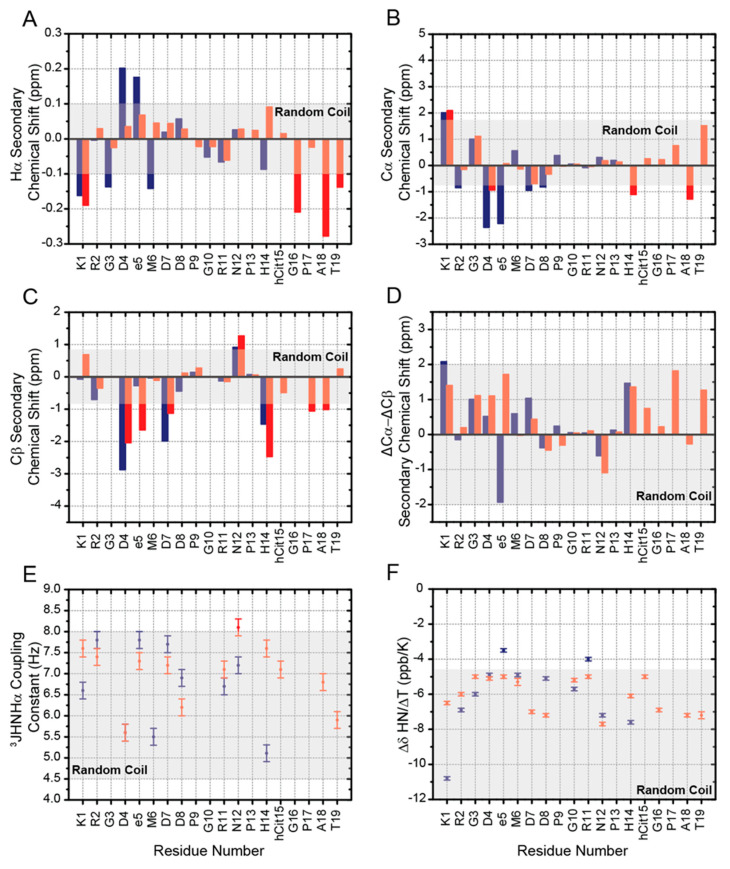
Secondary structure propensity of RGDechi1-14 and ψRGDechi. Hα (**A**), Cα (**B**) and Cβ (**C**) secondary chemical shifts analysis of RGDechi1-14 (blue) and ψRGDechi (red). The per-residue ΔδCα−ΔδCβ difference (**D**) for both peptides is also reported. The light grey rectangles in the A–D panels indicate the cut-off values for secondary structures identification as proposed by Wishart and Marsh (see materials and methods). (**E**) ^3^JHNHα coupling constants measured for RGDechi1-14 (blue) and ψRGDechi (red). Random coil values are also reported (light grey rectangle) (**F**) Amide temperature coefficients (ΔδHN/ΔT) versus the residue number for RGDechi1-14 (blue) and ψRGDechi (red). The light grey rectangle indicates values below the −4.6 ppb/K (light grey) that are consistent with the absence of amide proton hydrogen bonding. In the panels E and F, the error bars are also included.

To explore the conformational features of RGDechi1-14 and ψRGDechi peptides, we analyzed the chemical shifts, which are sensitive probes for the structural properties around the corresponding nuclei and, therefore, for the conformational ensemble sampled by the peptides in solution. In particular, the backbone chemical shifts are delicate reports of local polypeptide secondary structure, representing an average of multiple populations and, therefore, allowing the detection of minor contributions. First, for RGDechi1-14 and ψRGDechi peptides, the secondary chemical shifts (Δδ) of Hα, Cα, and Cβ nuclei (Figure 3A–C), which are defined as the difference between the observed chemical shift (δobs) and the residue specific random or statistical coil value (δrandom coil), have been used to detect and quantify secondary structure elements. The random coil chemical shifts were estimated from the amino acid sequence by using the approach defined by Poulsen and co-workers [34,35]. In the case of RGDechi1-14, Hα, and Cα chemical shifts significantly deviate from the random coil values for most of the amino acids situated inside the RGD cycle (Lys1, Asp4 and DGlu5) with the Δδ values alternating from positive to negative along the cycle, whereas for the residues located at the C-terminal tail of the peptide, the observed Hα and Cα shifts fall within the spectral region associated with random coil conformations. On the contrary, in the case of ψRGDechi, Hα and Cα chemical shifts slightly diverge from the coil values along whole peptide sequence including the region flanking the RGD sequence. Overall, the secondary chemical shift analysis suggests that both peptides do not adopt any preferential conformation. To further confirm these structural findings, we evaluated the Cβ secondary chemical shifts (Figure 3C) and we calculated the difference between Cα and Cβ secondary shifts (ΔδCα − ΔδCβ), a common way to report secondary chemical shifts [36] (Figure 3D). Consecutive positive (ΔδCα–ΔδCβ) values above +2 ppm indicate α-helix conformation, whereas negative values below −2 ppm are indicative of β-strand or extended structure [36]. As illustrated in Figure 3D, for the RGDechi1-14 peptide as well as for ψRGDechi, ΔδCα − ΔδCβ values are within the ±2 ppm range for the whole peptide amino acid sequence indicating the lack of stable secondary structures. In addition, we analyzed the amide proton chemical shifts by inspection of the 2D 1H-15N HSQC experiment acquired for RGDechi1-14 and ψRGDechi, respectively. As illustrated in Figure S2A,B, for both peptides the 2D 1H-15N HSQC spectrum shows narrow dispersion for most of the detected resonances in both dimensions indicating, according to the Hα, Cα, and Cβ secondary chemical shift analysis, that RGDechi1-14 and ψRGDechi peptides mainly adopt an unstructured conformation. Notably different from ψRGDechi, the RGDechi1-14 peptide the amide proton of the residue Lys1 shows a significant downfield shift that may be due to the presence of a hydrogen bond or to ring current effects of neighboring aromatic residues [37,38,39]. After that, we explored the Xaa-Pro peptide bond configurations by evaluating the difference between Cβ and Cγ chemical shifts (Δβγ = δCβ − δCγ) for all proline residues included in the peptide sequence of ψRGDechi and RGDechi1-14. In fact, several studies [40,41,42] have demonstrated that prolyl residues with cis peptide bonds typically have Cβ and Cγ chemical shifts around 35 and 25 ppm with a Δβγ difference around 10 ppm, whereas proline residues having trans peptide bonds present Cβ shifts around 32 ppm and Cγ resonances at 27 ppm with a Δβγ of about 5 ppm. As reported in Table S5, the two (Pro9 and Pro13) and three proline (Pro9, Pro13, and Pro17) residues in RGDechi1-14 and ψRGDechi, respectively, have a chemical shift difference of Δβγ~5 ppm, indicating that they present a trans Xaa-Pro peptide bond configuration. This latter finding was further confirmed by evaluating the chemical shifts using the Promega software [43] (Table S5). In agreement with the Cβ and Cγ chemical shift analysis, the 2D [1H-1H] ROESY spectra shows strong ROE cross peaks between one or both of the prolyl Hδ2 and Hδ3 and the Hα of the previous residues for the residue pairs: D8-P9/N12-P13 in the case of RGDechi1-14 and D8-P9, N12-P13 and G16-P17 for ψRGDechi (Figure S3A,B). Finally, we also estimated the population of the Xaa-Pro peptide bonds by evaluating, in the 1H-13C CT HSQC spectrum acquired for both peptides by natural abundance NMR spectroscopy, the intensity of signals belonging to the cis and trans form (Figure S3C). This analysis indicates for all Xaa-Pro peptide bonds an average cis population minor than 10% and 5% for RGDechi1-14 and ψRGDechi, respectively. Overall, the chemical shifts evaluation, as well as ROEs and intensity profile analysis, indicates that both peptides in solution sample an ensemble of interconverting transient conformers lacking specific organized structure with the proline residues presenting mainly a trans Xaa-Pro peptide bond configuration.

### 2.3. ^3^JHNHα Coupling Constants, HN Temperature Coefficients and NOEs Evaluation

Torsion angles φ and ψ are key conformational parameters to define the backbone conformation of a polypeptide chain [44]. In solution, these dihedral angles can be experimentally measured by NMR via scalar coupling constants, which in case of 3J can be related to specific torsion angles using the Karplus equation [45,46]. Vicinal (three bond) HN-Hα coupling constants (^3^JHNHα) are the most useful for the identification of secondary structure elements [47,48]. Notably, when the peptide adopts multiple conformations and there is a rapid interconversion (fast exchange on the NMR timescale) between them, the measured coupling constants represent a population weighted average of the conformers that build up the conformational ensemble [49]. The ^3^JHNHα coupling constants measured for RGDechi1-14 and ψRGDechi (Tables S6 and S7) are reported in Figure 3E and were found to be in the range of 5.5–7.8 Hz for RGDechi1-14 and 5.6–8.1 Hz for ψRGDechi. Then, we analyzed HN temperature coefficients (ΔδHN/ΔTs) reporting the dependence of chemical shift on temperature. In the case of structured peptides or proteins, amide protons usually display negative ΔδHN/ΔT values that depend on solvent exposure or hydrogen bond formation. In particular, ΔδHN/ΔTs more negative than −4.6 ppb/K are commonly observed for amide protons in peptide-solvent hydrogen bonds [50], whereas those less negative than −4.6 ppb/K are associated with intramolecular hydrogen bonds [50]. Instead, positive ΔδHN/ΔTs are due either to the conformational exchange or to the presence of aromatic amino acids [51,52]. The ΔδHN/ΔTs measured for RGDechi1-14 and ψRGDechi are reported in Figure 3F. In the RGDechi1-14 peptide, all residues within the RGD cycle show temperature coefficients more negative than −4.6 ppb/K, indicating large chemical shift changes inconsistent with the presence of hydrogen bonds, and the temperature coefficient for DGlu5 (ΔδHN/ΔT = −3.5 ppb/K) falls within the range expected for residues involved in hydrogen bond. In the C-terminal part of RGDechi1-14, Arg11 shows a temperature coefficient less negative than −4.6 ppb/K (ΔδHN/ΔT = −4.0 ppb/K), suggesting weak hydrogen bonding or a mixture of hydrogen-bonded and solvent-exposed amide protons, while all the other residues display ΔδHN/ΔT values more negative than −4.6 ppb/K, indicating the absence of intra-molecular hydrogen bonds. On the contrary, all residues of the ψRGDechi peptide sequence, including those flanking the RGD motif, exhibit amide proton temperature coefficients in the range from −5.0 to −7.2 ppb/K, demonstrating that the amide protons are involved in peptide–solvent hydrogen bonds. In addition to the insights on the conformational preferences of RGDechi1-14 and ψRGDechi obtained by evaluating chemical shifts, amide proton temperature coefficients, and 3JHNHα coupling constants, we exploited the structural and dynamical information provided by 2D [1H-1H] NOESY and 2D [1H-1H] ROESY spectra. Due to the peptide molecular tumbling that modulate the evolution and the sign of the NOE, the 2D [1H-1H] NOESY spectra of both peptides showed few and very weak intra-and inter-residue NOEs. On the contrary, 2D [1H-1H] ROESY spectra of RGDechi1-14 and ψRGDechi revealed only sequential (R = 1) and medium range (R < 5) ROE connectivities, indicating that both peptides do not adopt a compact folded structure. Altogether, ^3^JHNHα coupling constants, HN temperature coefficients, and ROESY cross-peak maps, in agreement with the chemical shifts analysis, clearly demonstrate that RGDechi1-14 and ψRGDechi peptides explore an ensemble of random coil lacking well-formed secondary structure elements.

### 2.4. Structural Comparison of RGDechi1-14 and ψRGDechi versus RGDechi

**Figure 4:**
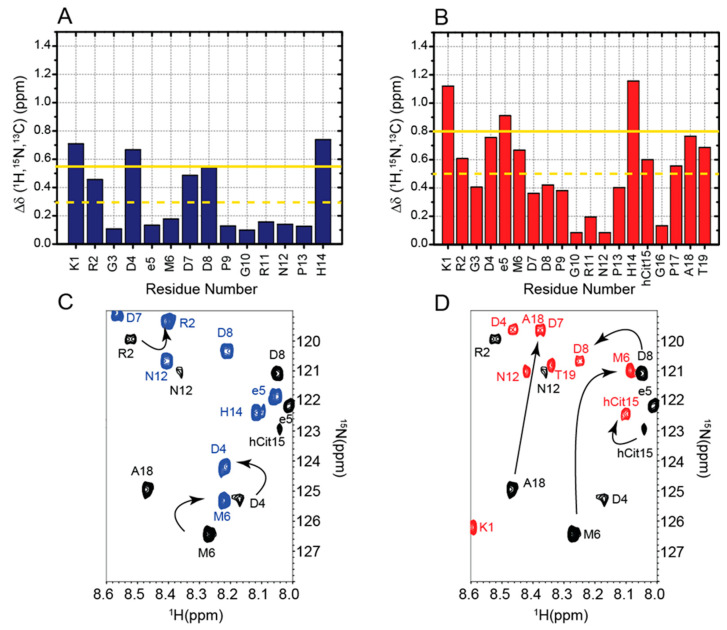
Structural effects of the chemical modifications in RGDechi1-14 and ψRGDechi. (**A**,**B**) Plot of combined 1H, 13C, and 15N chemical shifts perturbation as a function of the residue number of RGDechi1-14 and ψRGDechi, respectively. The CSP analysis for both peptides was performed using as reference the chemical shifts reported for the wild-type RGDechi in a previous publication (see main text). The yellow line indicates the average CSP (CSPavg), whereas the yellow dashed line reports the CSPavg + SD (standard deviation). (**C**,**D**) Overlay of the 1H-15N HSQC experiments of RGDechi1-14 (blue) and ψRGDechi (red) with the spectrum recorded for the wild-type RGDechi.

To investigate the conformational variations induced by the chemical modifications, we simultaneously compared all 1H, 15N, and 13C chemical shifts assigned for the backbone and side chains of RGDechi1-14 and ψRGDechi with those previously reported for the wild-type RGDechi (Figure 4A–D).

In the case of RGDechi1-14, the removal of the last five residues results in significant changes in the [1H-15N] HSQC and [1H-13C] HSQC single bond correlation spectra (Figure 4A). In particular, significant backbone and side-chain chemical shift perturbations (Δδ 1H,15N,13C) larger than the average value + SD (standard deviation) were observed for Lys1 and Asp4 located at the N-terminal part of the peptide within the RGD cycle, for Asp8 situated in the middle region of RGDechi1-14, and for the last C-terminal residue, His14 (Figure 4C). In the case of ψRGDechi, upon reduction of the peptide bond between Pro16 and Gly17, larger chemical shift variations (Δδ 1H,15N,13C ≥ average value + SD) were observed for Lys1, DGlu5, and His14, whereas significant small differences (Δδ 1H,15N,13C ≥ average value) were detected for Asp4, Met6, hCit15, Pro17, Ala18, and Thr19 (Figure 4D). Notably, in both cases residues situated inside the RGD cycle that are far beyond the chemical modification sites show substantial conformational variations with respect to the wild-type RGDechi. Our data demonstrate that the chemical modifications introduced in the RGDechi1-14 and ψRGDechi peptides induce long-range structural rearrangements, modifying the hydrogen bond-mediated structural coupling observed in RGDechi between the RGD moiety and the C-terminal tail peptide [16,29] that play a crucial role in the integrin recognition mechanism.

### 2.5. RGDechi1-14 and ψRGDechi Backbone Motions

**Figure 5:**
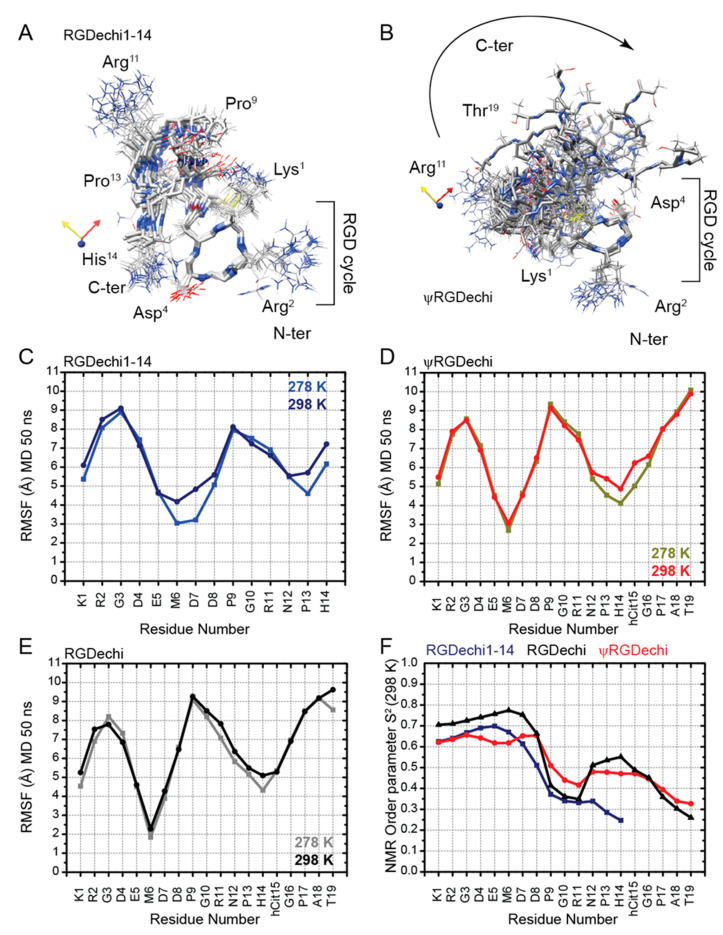
Picosecond to nanosecond backbone dynamics of RGDechi1-14 and ψRGDechi. (**A**,**B**) RGDechi1-14 and ψRGDechi conformational ensembles obtained after cluster analysis of the 10 ns Molecular dynamics simulation trajectories performed at 278 K. Each ensemble reports the 15 representative conformers of the most populated clusters. The conformers were aligned with respect to the RGD cycle. (**C**–**E**) root mean square fluctuation (RMSF) (Å) values obtained from the 50 ns MD simulations performed at 278 and 298 K for RGDechi1-14 (**C**) and ψRGDechi (**D**) and RGDechi (**E**), respectively. (**F**) Comparison of the predicted H-N model-free order parameters (S^2^), as reported in the materials and methods section, from backbone and Cβ chemical shifts for RGDechi1-14 (blue), ψRGDechi (red), and RGDechi (black).

**Figure 6:**
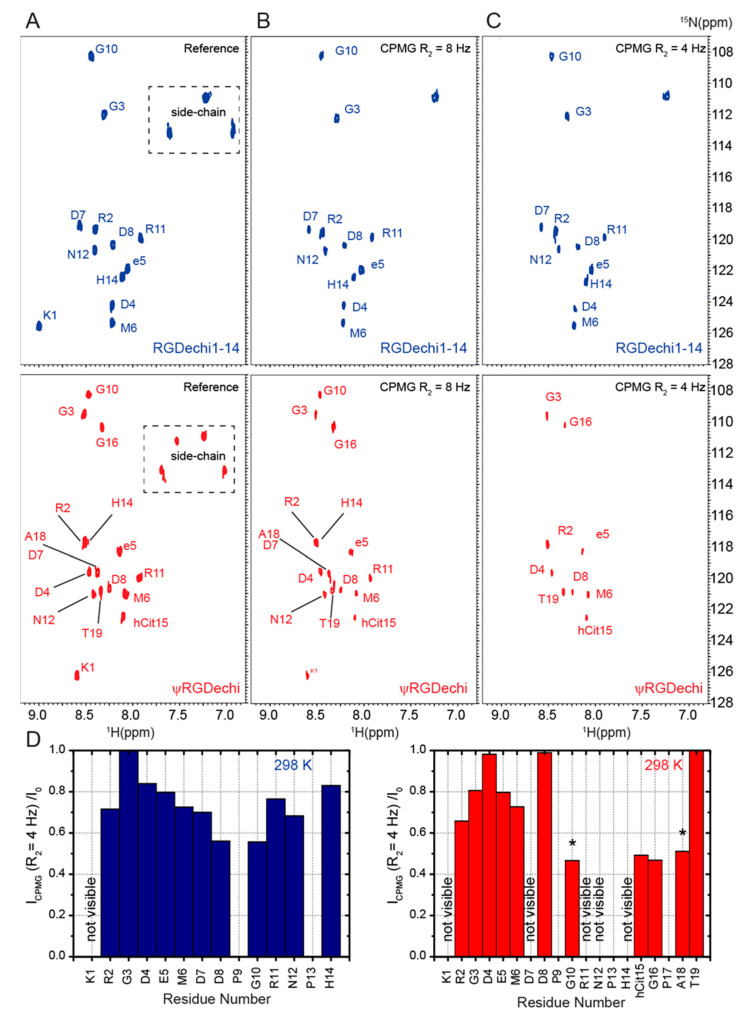
Microsecond to millisecond backbone motions of RGDechi1-14 and ψRGDechi. (**A**–**C**) Comparison of the reference 1H-15N HSQC experiments recorded for RGDechi1-14 and ψRGDechi with the filtered 1H-15N HSQC spectra of both peptides acquired using a relaxation-compensated CPMG period of 125 ms (R_2_ = 8 Hz) (**B**) and 250 ms (R_2_ = 4 Hz) (**C**,**D**) Normalized intensity ratios between the NMR signal observed for RGDechi1-14 (left) and ψRGDechi (right) at 298 K in the T_2_-filter 1H-15N HSQC experiments (CPMG R_2_ = 8 Hz) (I_CPMG_) with the values observed in the 1H-15N HSQC spectra acquired without any filter (I_0_). The asterisk indicates the residues exhibiting low signal to noise ratio.

To investigate the RGDechi1-14 and ψRGDechi conformational dynamics, we explored 15N backbone motions on the picosecond (ps) to millisecond (ms) timescale by using natural-abundance NMR experiments and we probed the nanosecond (ns) conformational flexibility using MD simulations. At first, we generated conformational ensembles reproducing nanosecond intrinsic motions of RGDechi1-14 and ψRGDechi (Figure 5A,B) at 278 and 298 K. In particular, we performed a series of MD simulations at 298 K of 10 ns each, collecting up to 50 ns of simulation time. Then, we investigated the backbone conformational dynamics of each peptide by calculating the per-residue Cα root mean square fluctuation (RMSF) from the 50 ns MD ensembles obtained at the two different temperatures (Figure 5C,D). In the case of RGDechi1-14, the RMSF values show that the residues from Lys1 to DGlu5 (average RMSF1-5_avg_ (298 K) = 7.09 ± 1.38 Å; RMSF1-5_avg_ (278 K) = 6.89 ± 1.49 Å) forming the RGD cycle exhibit enhanced atomic fluctuations with respect to the rest of the peptide from Met6 to Hist14 (RMSF6-14_avg_ (298 K) = 6.11 ± 1.05 Å; RMSF6-14_avg_ (278 K) = 5.55 ± 1.41 Å). For ψRGDechi, the analysis of the RMSFs indicates that the N-term RGD moiety (Lys1-DGlu5) (RMSF1-5_avg_ (298 K) = 6.65 ± 1.35 Å; RMSF1-5_avg_ (278 K) = 6.63 ± 1.44 Å) is only minimally more rigid than the C-terminal tail (Met6-Thr19) (RMSF6-19_avg_ (298 K) = 6.75 ± 1.58 Å; RMSF6-19_avg_ (278 K) = 6.53 ± 1.91 Å). In addition, to fully describe the impact of the chemical modifications on the nanosecond-scale backbone dynamics, we generated a 50 ns MD conformational ensemble for the RGDechi peptide. As reported in Figure 5E, RMSF values of the wild-type peptide indicate that, as observed for ψRGDechi, the RGD cycle (RMSF1-5_avg_ (298 K) = 6.41 ± 1.18 Å; RMSF1-5_avg_ (278 K) = 6.30 ± 1.41 Å) presents less conformational fluctuations than the region (Met6-Thr19) located at the C-terminal (RMSF6-19_avg_ (298 K) = 6.79 ± 1.55 Å; RMSF6-19_avg_ (278 K) = 6.47 ± 1.77 Å). Interestingly, the comparison of the RMSF values obtained for the three peptides clearly indicate that the RGD cycle backbone dynamics on the nanosecond timescale are strongly influenced by the chemical modifications introduced at the C-terminal tail. In fact, the removal of the last five residues (RGDechi1-14) and the reduction of the amide bond Pro16-Gly17 (ψRGDechi) induce a remarkable increase in conformational flexibility of the RGD cycle most likely affecting the binding selectivity for α_v_β_3_ integrin. Successively, we integrated the dynamics information reported by the backbone chemical shifts (Figure 5F) with data obtained by measuring a couple of T2-filter [1H–15N] HSQC experiments, using two relaxation-compensated CPMG periods (125 (R_2_ = 8 Hz) and 250 (R_2_ = 4 Hz) ms) (for details see materials and methods) (Figure 6A–C). Interestingly, as reported in Figure 6C, the T_2_-filter [1H–15N] HSQC experiments acquired for the two RGDechi-derived peptides using a filter delay of 250 ms (R_2_ = 4 Hz) for RGDechi1-14 report all the expected 1H-15N cross peaks with the exception of Lys1. On the contrary, for ψRGDechi the 1H-15N signal loss were observed for residues Lys1, Asp7, Arg11, Asn12, and His14 indicating that these residues are characterized by a 15N R_2_ (1/T_2_) auto-relaxation rate constant faster than 4 Hz (Figure 6D). Overall, T2-filter [1H–15N] HSQC data analysis clearly indicates that the two peptides show different conformational dynamics on the millisecond timescale. Finally, to provide a complete description of RGDechi1-14 and ψRGDechi backbone dynamics, we explored internal flexibility on the ps time scale by evaluating the model-free order parameter (S^2^) at 298 K for the backbone amide group. In both cases, as expected, S^2^ order parameter values indicate that the residues encompassing the cycle region (S^2^_avg_ = 0.66 ± 0.03 for RGDechi1-14 and S^2^_avg_ = 0.73 ± 0.02 for ψRGDechi) are characterized by a higher degree of rigidity than the C-terminal tail (S^2^_avg_= 0.41 ± 0.12 for RGDechi1-14 and S^2^_avg_ = 0.48 ± 0.13 for ψRGDechi) in the ps time scale. Overall, the dynamical characterization of RGDechi1-14 and ψRGDechi demonstrates that the two peptides present in solution an elevate conformational heterogeneity with the C-terminal region, showing a higher degree of flexibility than the RGD cycle in both ns-to-ps and µs-to-ms time scale. Additionally, the comparison of the RGDechi1-14 and ψRGDechi backbone dynamics data with those obtained for the wild-type RGDechi indicates that the C-terminal tail modulates the conformational flexibility of the RGD moiety and it may represent a hotspot region for fine tuning integrin binding mechanism.

### 2.6. Conformational Ensemble of RGDechi1-14 and ψRGDechi Peptides

**Figure 7:**
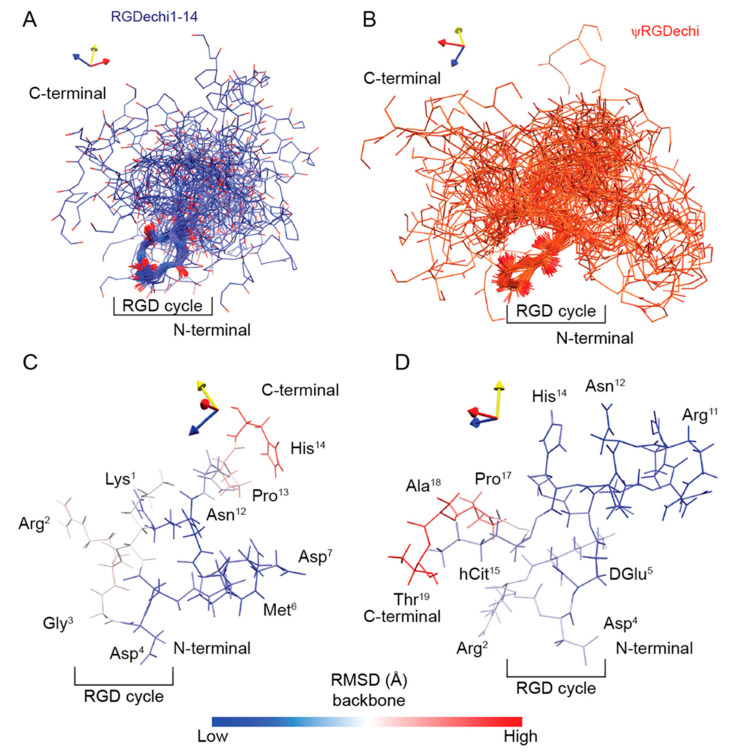
Conformational ensembles of RGDechi1-14 and ψRGDechi. (**A**,**B**) Conformational ensembles obtained integrating MD simulations data with experimental NMR chemical shifts. Each structural ensemble was generated by selecting the 50 structures having the lowest global CS-RMSD function. In both ensembles the conformers were aligned with respect to the RGD cycle region from Lys1 to DGlu5. (**C**,**D**) Backbone RMSD values reported on the representative structure (conformer with the lowest CS-RMSD function) for RGDechi1-14 and ψRGDechi peptides.

The high degree of conformational dynamics of fully or partially disordered peptides needs an ensemble of fairly heterogeneous conformers for its representation. Therefore, to describe the structural ensemble of RGDechi1-14 and ψRGDechi, we applied a computational approach based on experimental NMR chemical shifts (CS) (Figure S4A,B). Firstly, we evaluated the MD ensemble (5000 conformers) generated for RGDechi1-14 and ψRGDechi as reported above through cluster analysis. For each peptide, this latter procedure resulted in a representative cluster containing 391 and 435 conformers for RGDechi1-14 and ψRGDechi, respectively (Figure S4A,B). Secondly, in order to generate a conformational ensemble able to provide a rigorous description of the solution NMR data for each peptide, we analyzed the 50 ns MD clusters using the experimental 1H,15N and 13C chemical shifts assigned for the two RGDechi-derived peptides (Figure S4A,B). In particular, we used PPM software [53,54] as a chemical shift prediction tool to back-calculate the backbone and side chain CSs from each conformer of the RGDechi1-14 and ψRGDechi MD clusters. Then, we compared the measured chemical shifts with those predicted for each conformer using a global chemical shift root mean square deviation (global CS-RMSD) function. As reflected by the global CS-RMSD values, the CS-based selection protocol allowed for the identification of the representative conformers, proving a reasonable description of the NMR experimental data for both peptides. Thirdly, the 50 structures having the lowest global RMSD function were selected to generate the final representative conformational ensemble (MD ensemble 50×) for RGDechi1-14 and ψRGDechi, respectively (Figure 7A,B). Interestingly, for the two peptides the MD ensemble 50× provided a significant improvement of the global CS-RMSDs to 4.58 ppm for RGDechi1-14 and 5.76 ppm for ψRGDechi indicating that, in both cases, the 50 conformers better describe the conformational space than any of them separately (Figure S5A,B). Moreover, we also evaluated for each peptide two additional conformational ensembles named MD ensemble2 50× and MD ensemble3 50×; the former was generated by considering the 50 conformers with the highest global CS-RMSD, while the latter was obtained by randomly selecting 50 conformers. As reported in Figure S5A,B, for both peptides the MD ensemble2 50× and MD ensemble3 50× did not provide any improvement of the global CS-RMSDs. Then, to further validate the MD ensemble 50× obtained for each peptide, we fitted the experimental ^3^JHNHα coupling constants to the values back-calculated from the conformational ensemble. The measured ^3^JHNHαs provided excellent fits for both peptides with correlation coefficients of 0.83 and 0.86 for RGDechi1-14 and ψRGDechi, respectively (Figure S6A,B). Overall, the data show that the generated and validated MD ensemble 50× provides an accurate description of the NMR parameters for both peptides. In agreement with the NMR and MD backbone dynamics characterization, the RMSD values analysis of the obtained MD ensemble 50× conformational ensembles indicate that in both peptides the N-terminal part, containing the RGD cycle, presents less degree of conformational flexibility than the C-terminal tail which is characterized by a high structural heterogeneity (Figure 7C,D)

### 2.7. Elucidation on the Recognition Mechanism of α_v_β_3_ Integrin by ψRGDechi

In order to obtain insights on the relationships between the structure, dynamics, and function of ψRGDechi, we explored the molecular mechanism by which the peptide recognizes the α_v_β_3_ integrin. The structural model of the ψRGDechi/α_v_β_3_ obtained by using experimental and computational data has demonstrated that the peptide in the complex adopts a relatively compact conformation [33]. Therefore, we set out to understand whether the conformational ensemble sampled in solution by ψRGDechi in absence of the binding partner contains conformers having structural similarities with the peptide in the bound form. We compared the 1H, 15N, and 13C chemical shifts observed for ψRGDechi in the free form to the values back-calculated from the conformation adopted by the peptide in the complex with α_v_β_3_ integrin. As indicated by the Q factor values in the range of 0.13–0.36 (Figure S7A–C), as well as by the per-residue cumulative chemical shift differences (Figure S7D), the experimental shifts measured for the ψRGDechi in the free form cannot be described by the structure that the peptide adopts upon binding to α_v_β_3_ integrin, indicating that the whole ψRGDechi experiences significant structural rearrangements upon binding. Overall, the data suggest that the recognition of α_v_β_3_ by ψRGDechi is mainly driven by an induced fit binding mechanism.

## 3. Materials and Methods

### 3.1. Peptides Preparation

The peptides RGDechi1-14 (amino acid sequence c[K-R-G-D-e]-M-D-D-P-G-R-N-P-H) and ψRGDechi (amino acid sequence c[K-R-G-D-e]-M-D-D-P-G-R-N-P-H-hCyt-G-P-A-T) were synthetized as previously reported [29,33].

### 3.2. NMR Experiments

NMR measurements were performed at 298 K by using a Bruker AVIII HD 600-MHz spectrometer equipped with a triple-resonance Prodigy N2 cryoprobe and a z-axis pulse field gradient. All NMR samples were prepared by dissolving the RGDechi1-14 or ψRGDechi peptides in 200 μL of Hank’s balanced salt solution (HBSS) buffer with pH 7.4 and 10% ^2^H2O in a 3 mm NMR tube. The final concentration was 0.4 mM for both peptides. The chemical shift assignments of RGDechi1-14 and ψRGDechi were obtained by acquiring and analyzing the following homonuclear and natural abundance heteronuclear NMR spectra:

(i) The 2D [1H-1H] TOCSY (total correlation spectroscopy) [55], 2D [1H-1H] NOESY (nuclear overhauser effect spectroscopy) [56], and 2D [1H-1H] ROESY (rotating frame overhauser effect spectroscopy) [57] data were acquired using 32 scans per t1 increment, a spectral width (SW) of 6001.30 Hz along both t1 and t2, 2048 × 256 complex points in t2 and t1, and a relaxation delay of 3.0 s. In all NMR experiments reported above, the water suppression was achieved using the Watergate pulse sequence with the application of gradients using double echo. The 2D [1H-1H] TOCSY experiments were acquired by homonuclear Hartman–Hahn transfer by a MILEV17 mixing sequence with a mixing time (tm) of 70 ms and 10 KHz spin-lock field strength; The 2D [1H-1H] NOESY spectra were recorded using a mixing time of 250 ms and 2D [1H-1H] ROESY were carried out with a cw spin-lock field strength of 4 kHz, a mixing time of 250 ms, and a relaxation delay of 5.0 s. All homonuclear 2D experiments were apodized with a square cosine window function and zero fill to a matrix of size 4096 × 1024 before Fourier transform and baseline correction.
(ii) Heteronuclear natural-abundance 2D [1H–15N] HSQC (heteronuclear single quantum coherence) experiments were performed with 880 scans per t1 increment, SW of 1581.32 and 6001.30 Hz along t1 and t2, respectively, 2048 × 128 complex points in t2 and t1, respectively, and a 1.0 s relaxation delay. In particular, the 2D [1H–15N] HSQC pulse sequence was optimized as previously reported [16] using a Bruker standard gradient echo–antiecho pulse program [58]. Two-dimensional [1H–15N] HSQC spectra were apodized with a square cosine window function and a zero filling to a matrix of size a 2048 × 128 before Fourier transform and baseline correction. Natural-abundance 2D [1H–13C] HSQC spectra were acquired with 880 scans per t1 increment, SW of 5130.94 Hz along t1 and 6001.30 Hz along t2, 2048 × 200 complex points in t2 and t1, respectively, and a relaxation delay 1.0 s. The 2D constant time (CT) [1H–13C] HSQC pulse sequence was optimized as reported is a previous publication [16]. Two-dimensional CT [1H–13C] HSQC were acquired using a heteronuclear coupling constant (JXH) of 145 Hz, CT period of 26.6 ms, and shaped pulses for all 180° pulses on f2 channel with decoupling during f1 acquisition. Two-dimensional CT [1H–13C] HSQC spectra were apodized with a square cosine windows function and zero fill to a final matrix of 4096 × 4096 before Fourier transform and baseline correction.

The description of RGDechi1-14 and ψRGDechi backbone dynamics was performed at 298 K and 600 MHz by measuring natural-abundance NMR spectra as reported below:

(i) Two-dimensional T2-filter [1H–15N] HSQC experiments were carried out using 880 scans per t1 increment, SW of 1581.32 Hz along t1 and 6001.30 Hz along t2, 2048 × 128 complex points in t2 and t1, respectively, and 1.0 s relaxation delay. In particular, for each peptide, the dynamics characterization was performed by two 2D T2-filter [1H–15N] HSQC spectra acquired using different relaxation compensated CPMG (Carr–Purcell–Meiboom–Gill) sequence periods (125 and 250 ms). Notably, in this [1H–15N] HSQC-based experiment only the 1H-15N cross-peaks of the residues for which the 15N transversal relaxation time (T2) was longer than the applied filter were observable. The pulse sequence was optimized as previously reported [16]. Two-dimensional T2-filter [1H–15N] HSQC spectra were apodized with a square cosine window function and zero filling to a matrix of size 4096 × 1024 before Fourier transform and baseline correction.

To measure amide temperature coefficients (ΔδHN/ΔT) and 3JHNHα vicinal scalar coupling constants, 1D 1H NMR spectra were carried out with a SW of 6001.30 Hz, 1.0 s relaxation delay, and 64 K and 256 K data points for acquisition and transformation, respectively.

The structural and dynamical characterization was conducted using well-resolved ROE signals. In particular, ROEs analysis was mainly performed using cross-peaks related to the amide protons region in which we did not observe any 1H signal from the buffer components. NMR signals characterized by a strong overlap with buffer resonances were excluded from the analysis. All 2D NMR spectra were processed by NMRPipe [59] and analyzed using NMRFAM-SPARKY [60] and CARA [61] software.

### 3.3. Chemical Shifts Analysis

Using 4,4-dimethyl-4-silapentane-1-sulfonic acid (DSS) as a standard, 1H chemical shifts were directly calibrated, whereas 15N and 13C shifts were indirectly referenced. Hα, Cα, and Cβ secondary chemical shifts were calculated as the difference between the observed shift and the value predicted for the random coil conformation. The random coil reference values were estimated from the peptide sequence using the approach defined by Poulsen and co-workers [34,35]. Secondary chemical elements were identified using the reference values defined by Wishart [62] and Marsh [36]. In the case of ψRGDechi, the homocitruline (hCit) was treated as a lysine because it is different from lysine only in the replacement of the amine group with a carbamylate one at the end of the long aliphatic side chain. The statistical probability of trans/cis Xaa-Pro peptide bond was estimated from the amino acid sequence of the peptides, the proline chemical shifts, and the backbone chemical shifts of neighboring residues using the approach implemented in the Promega software [43]. In addition, for each proline, the Xaa-Pro peptide bond configuration was investigated by evaluating the difference between Cβ and Cγ chemical shifts (Δβγ = δCβ − δCγ) using the Δβγ reference values reported by Shubert et al. [41]. The 1H, 15N, and 13C chemical shift predictions for each conformer extracted from the conformational ensemble obtained by molecular dynamics simulations (see above) were performed using the software PPM_ONE [54]. The quality or Q factor was calculated using the equation reported below:

Q = rms (Δδobs − Δδpred)/rms(Δδobs)

where δobs is the measured chemical shift and δpred is the back-calculated chemical shift on the base of the 3D structure of the investigated model.

### 3.4. Amide Temperature Coefficients and 3JHNHα Scalar Couplings Analysis

Temperature coefficients (Δδ/ΔT; ppb/K) for amide protons were obtained for each residue from the gradient of the linear plot of HN chemical shift versus temperature. Amide proton chemical shifts for RGDechi1-14 and ψRGDechi peptides were obtained from a series of 1D spectra acquired at 298, 300, 302, 304, 306, 308, and 310 K. The 3JHNHα couplings of RGDechi1-14 and ψRGDechi were measured at 298 K from 1D 1H NMR spectra.

### 3.5. Molecular Dynamics Simulations

Starting structural models of RGDechi, RGDechi1-14, and ψRGDechi were built by the 3D builder package of Maestro suite (Schrödinger release, April 2019, LLC, New York, NY, USA) by selecting the extended secondary structure. Next, chemical modifications were manually modeled and the obtained linear peptides were refined and energy minimized prior to MD simulations. RGDechi, RGDechi1-14, and ψRGDechi’s parameters were estimated using the Antechamber suite [63] and virtually analyzed and refined using the Leap module. The Amber v18 [64] MD simulation package was used to perform in-silico simulations by applying Amber-ff14SB force field [65]. Each peptide was centered in a triclinic box and solvated by a 10 Å shell of explicit TIP3P water [66] and counter ions (Na+) were added to neutralize the systems. Energy minimization was carried out by the steepest descent method and then 1 ns equilibration phase was performed where heavy atoms were position restrained by a harmonic potential, unrestrained systems were simulated in a NPT ensemble using the Langevin equilibration scheme to keep the temperature constant at 298 K or 278 K and the pressure constant at 1 atm. Electrostatic forces were evaluated by the particle mesh Ewald method [67] and Lennard–Jones forces by a cutoff of 10 Å. All bonds involving hydrogen atoms were constrained using the SHAKE algorithm [68]. Periodic boundary conditions were imposed in all three dimensions and the time step was set to 2 fs. Production runs of 10 ns were obtained at constant temperature of 298 K and 178 K and structures were recorded every 10 ps collecting a total of 2000 snapshots along the simulation time. In particular, to enhance conformational sampling of RGDechi, RGDechi1-14, and ψRGDechi peptides we ran five independent replicas of 10 ns at 298 K and 278 K by applying different random initial velocities (random seed ig = −1 in amber input settings). The conformational ensemble obtained for each peptide was clustered using the protocol defined by Kelley et al. [69]. This procedure generated 391, 435, and 427 structural families for RGDechi1-14, ψRGDechi, and RGDechi, respectively. Successively, the representative cluster structures were analyzed using the experimental 1H, 15N, and 13C chemical shifts. In particular, for each representative cluster structure the chemical shifts were back-calculated using PPM and PPM_ONE software [53,54] and then the predicted values were compared against the experimental shifts using a global root mean square deviation (global CS-RMSD) value (Σ RMSDsCα, Cβ, HN, Hα, N, H side chain). The final conformational ensemble for each peptide was generated by selecting the 50 structures showing the lowest global CS-RMSD value. All selected MD conformers were visualized and analyzed using the following software: PyMOL [70], CHIMERA [71], PROCHECK-NMR [72], and WHAT-IF [73]. The 3JHNHα scalar coupling constants were estimated from MD simulations using the Karplus relation [45,46] by taking the average of the φ angles measured from the MD ensemble 50x. The parameters defined by Bax and co-workers were used as coefficients for the Karplus equation [74].

## 4. Conclusions

In this study, we have performed a structural and dynamical characterization using natural-abundance NMR and MD techniques of two RGDechi-derived peptides called RGDechi1-14 and ψRGDechi. A nearly complete 1H, 15N, 13C chemical shifts assignment has been obtained by exploring natural-abundance NMR methodologies using a particular strategy based on the combination of 2D homo- and heteronuclear experiments. Hα, Cα, and Cβ secondary chemical shifts, as well as ^3^JHNHα scalar coupling constants, HN temperature coefficients (ΔδHN/ΔTs), and ROE connectivities indicate that both peptides present a high degree of conformational flexibility in solution, especially within their C-terminal regions, without adopting any well-folded secondary structure and with all proline residues mainly in a trans Xaa-Pro peptide bond configuration. NMR and MD backbone dynamics characterization demonstrate that, in both peptides, the RGDechi cycle is slightly more rigid than the C-terminal tail. However, in RGDechi1-14, the removal of the last five residues induces a substantial increase in the conformational mobility of the RGD cycle with respect to the wild-type RGDechi. Interestingly, comparison of the backbone dynamics between ψRGDechi and RGDechi shows that the reduction of the peptide bond between Pro16 and Gly17 slightly increases the conformational flexibility of the RGD moieties. These findings, together with previously published serum stability and α_v_β_3_ binding studies, indicate that in the ψRGDechi the chemical modification drastically reduces the enzymatic degradation in serum while preserving the ability to recognize the α_v_β_3_ receptor when compared to the wild-type RGDechi without significantly altering the conformational proprieties of the peptide in solution [33]. Overall, taking into account previously reported data [16], our study clearly demonstrates that, in RGDechi, the hydrogen bond-mediated structural coupling between the N- and C-terminal regions modulates the conformational flexibility of the RGD cycle, that in turn plays a crucial role in the fine tuning of the integrin binding selectivity. Nevertheless, all these data may pave the way for the development of novel RGDechi-derived peptides by introducing chemical modifications at the C-terminal region that in turn modulate the flexibility of the RGD cycle in order to selectively bind specific members of the integrin family. In conclusion, our study demonstrates that the combination of NMR and MD techniques represents an efficient strategy for exploring the conformational space sampled in solution by peptides having a high degree of conformational heterogeneity.

## Data Availability

Not applicable.

## Supplementary Materials

The following supporting information can be downloaded at: https://www.mdpi.com/article/10.3390/ijms231911039/s1.
