# Supplementary material for: High-Resolution Conformational Analysis of RGDechi-Derived Peptides Based on a Combination of NMR Spectroscopy and MD Simulations
Source: Int J Mol Sci. 2022 Sep 20;23(19):11039. doi: 10.3390/ijms231911039 (PMC9569650; doi:10.3390/ijms231911039)

Supplementary Materials

Table S1. ^{1}H and ^{15}N chemical shifts assignment* of ψRGDechi in HBSS buffer (pH 7.4) at 298K on 600 MHz spectrometer.

Residue	H_N	***N***	$H\alpha$	$H\beta$	***Others***
Lys^{1}	8.59	126.2	4.13	1.85,1.76	γCH_2 1.51, 1.37<br>δCH_2 1.71<br>εCH_2 3.01
Arg^{2}	8.51	117.6	4.41	1.90, 1.85	γCH_2 1.61<br>δCH_2 3.20<br>εCH_2 7.53
Gly^{3}	8.52	109.5	4.15, 3.78		
Asp^{4}	8.46	119.6	4.62	2.84, 2.73	
DGlu^{5}	8.14	118.3	4.36	2.17, 2.13	γCH_2 2.49
Met^{6}	8.09	121.0	4.51	2.06, 1.86	γCH_2 2.60, 2.50<br>εCH_3 2.10
Asp^{7}	8.37	119.6	4.65	2.80, 2.69	
Asp^{8}	8.25	120.7	4.92	2.81, 2.62	
Pro^{9}			4.40	2.23, 1.80	γCH_2 1.99, 1.81<br>δCH_2 3.73
Gly^{10}	8.47	108.3	3.95, 3.88		
Arg^{11}	7.93	120.0	4.32	1.86, 1.73	γCH_2 1.63, 1.58<br>δCH_2 3.19<br>εNH 7.24
Asn^{12}	8.42	121.0	4.96	2.86, 2.74	δNH_2 7.70, 7.02
Pro^{13}			4.41	2.26, 1.99	γCH_2 2.03, 1.81<br>δCH_2 3.79

His[14]	8.51	117.7	4.70	3.31, 3.20	δ2H 6.97<br>ε1H 7.26
hCyt[15]	8.09	122.5	4.37	1.86, 1.76	γCH_2 1.36<br>δCH_2 1.50<br>εCH_3 3.02
Gly[16]	8.33	110.4	4.08,4.04		
Pro[17]			4.42	2.14, 1.80	γCH_2 2.07, 1.81<br>δCH_2 3.79
Ala[18]	8.38	119.7	4.11	1.56	
Thr[19]	8.34	120.9	4.20	4.25	γCH_2 1.20

*^{1}H ,^{15}N chemical shifts error values are ± 0.01 ppm and ± 0.4 ppm, respectively.

Table S2. ^{13}C chemical shifts assignment* of ψRGDechi in HBSS buffer (pH 7.4) at 298K on 600 MHz spectrometer.

Residue	***Cα***	***Cβ***	***Others***
Lys[1]	58.4	33.7	Cγ 25.1
			Cδ 29.1
			Cε 42.1
Arg[2]	56.1	30.4	Cγ 27.2
			Cδ 43.3
Gly[3]	46.5		
Asp[4]	53.8	39.0	
DGlu[5]	56.9	28.3	Cγ 33.5
Met[6]	55.6	32.7	Cγ 32.2
			εCH_3 17.1
Asp[7]	53.8	40.0	
Asp[8]	52.1	40.5	
Pro[9]	63.6	32.2	Cγ 27.1
			Cδ 50.7
Gly[10]	45.4		
Arg[11]	56.0	30.9	Cγ 27.2
			Cδ 43.4
Asn[12]	51.4	39.2	
Pro[13]	63.6	32.1	Cγ 27.3
			Cδ 49.7
His[14]	55.3	28.6	
hCyt[15]	56.6	33.7	Cγ 22.6

			Cδ 31.3
			εCH$_2$ 42.2
Gly[16]	44.7		
Pro[17]	63.9	31.2	Cγ 27.1
			Cδ 50.8
Ala[18]	51.3	18.1	
Thr[19]	63.6	70.4	Cγ 22.2

*^{13}C chemical shifts error values is ± 0.3 ppm

Table S3. ^{1}H and ^{15}N chemical shifts assignment* of RGDechi1-14 in HBSS buffer (pH 7.4) at 298K on 600 MHz spectrometer.

Residue	***H_N***	***N***	***Hα***	***Hβ***	***Others***
Lys[1]	8.99	125.6	4.16	1.80	γCH$_2$ 1.51, 1.44
					δCH$_2$ 1.69
					εCH$_2$ 3.00
Arg[2]	8.40	119.4	4.37	1.87, 1.68	γCH$_2$ 1.55
					δCH$_2$ 3.21
					εCH$_2$ 7.22
Gly[3]	8.31	112.0	4.15, 3.56		
Asp[4]	8.21	124.2	4.78	2.90, 2.65	
DGlu[5]	8.06	121.9	4.46	2.07, 1.90	γCH$_2$ 2.38
Met[6]	8.22	125.3	4.32	2.06, 1.99	γCH$_2$ 2.61, 2.56
					εCH$_3$ 2.10
Asp[7]	8.57	119.7	4.63	2.83	

Asp[8]	8.20	120.3	4.95	2.86, 2.62	
Pro[9]			4.43	2.30, 2.00	γCH_2 2.03
					δCH_2 3.78
Gly[10]	8.44	108.2	3.92, 3.85		
Arg[11]	7.92	119.9	4.31	1.86, 1.76	γCH_2 1.59
					δCH_2 3.20
					εNH 7.24
Asn[12]	8.42	120.7	4.96	2.83, 2.67	δNH_2 7.62, 6.94
Pro[13]			4.40	2.23, 1.91	γCH_2 1.96, 1.84
					δCH_2 3.75,3.70
His[14]	8.11	122.4	4.50	3.26, 3.13	δ2H 7.30
					ε1H 8.59

*^{1}H ,^{15}N chemical shifts error values are ± 0.01 ppm and ± 0.4 ppm, respectively.

Table S4. ^{13}C chemical shifts assignment* of RGDechi1-14 in HBSS buffer (pH 7.4) at 298K on 600 MHz spectrometer.

Residue	***Cα***	***Cβ***	***Others***
Lys[1]	58.4	32.9	Cγ 25.2
			Cδ 29.0
			Cε 42.2
Arg[2]	55.4	30.0	Cγ 27.3
			Cδ 43.4
Gly[3]	46.3		
Asp[4]	52.4	38.2	
DGlu[5]	54.6	29.7	Cγ 33.6

Met[6]	56.3	32.7	Cδ 32.0<br>εCH₃ 16.9
Asp[7]	53.5	39.2	
Asp[8]	51.6	39.9	
Pro[9]	64.0	32.1	Cγ 27.2 Cδ 50.7
Gly[10]	45.4		
Arg[11]	56.0	30.9	Cγ 27.1<br>Cδ 43.3
Asn[12]	51.5	38.9	
Pro[13]	63.6	32.1	Cγ 27.2<br>Cδ 50.7
His[14]	56.6	29.7	

*^{13}C chemical shifts error values is ± 0.3 ppm

Table S5. $^{13}C\beta$ and $^{13}C\gamma$ chemical shifts, their difference $\Delta\beta\gamma$ and predictions of *cis* Xaa-Pro likelihood for prolines of ψRGDechi and RGDechi1-14.

ψRGDechi				
Residue	***Cβ***	***Cγ***	***Δβγ***[a]	***Normalized likelihood for Pro to be in cis***[b]
Pro[9]	32.2	27.1	5.1	0.004
Pro[13]	32.1	27.3	4.8	0.016
Pro[17]	31.2	27.1	4.1	0.005
RGDechi1-14				
Pro[9]	32.1	27.2	4.9	0.002
Pro[13]	32.1	27.2	4.9	0.019

[a] Values of $\Delta\beta\gamma$ ($\Delta\beta\gamma = \delta[C\beta] - \delta[C\gamma]$) for prolines are function of the Xaa-Pro peptide bond conformation: $\Delta\beta\gamma$ Pro trans = 4.51 ± 1.17 and $\Delta\beta\gamma$ Pro cis = 9.64 ± 1.27.

[b]The predictions were performed by using the Promega server from the amino acid sequence and chemical shift data.

Table S6. ^{3}JNHα coupling constants of *ψ*RGDechi in HBSS buffer (pH 7.4) at 298K on 600 MHz spectrometer.

Residue	$^{\mathbf{3}}\boldsymbol{J_N H\alpha}$***(Hz)***
Lys^1	7.61
Arg^2	7.39
Gly^3	
Asp^4	5.64
$DGlu^5$	7.32
Met^6	
Asp^7	7.22
Asp^8	6.21
Pro^9	
Gly^{10}	
Arg^{11}	7.08
Asn^{12}	8.14
Pro^{13}	
His^{14}	7.58
$hCyt^{15}$	7.07
Gly^{16}	
Pro^{17}	
Ala^{18}	6.84
Thr^{19}	5.92

Table S7. ^{3}JHNHa coupling constants of RGDechi1-14 in HBSS buffer (pH 7.4) at 298K on 600 MHz spectrometer.

Residue	***$^3J_NH\alpha$(Hz)***
Lys[1]	6.57
Arg[2]	7.79
Gly[3]	
Asp[4]	5.57
DGlu[5]	7.75
Met[6]	5.48
Asp[7]	7.70
Asp[8]	6.87
Pro[9]	
Gly[10]	
Arg[11]	6.74
Asn[12]	7.18
Pro[13]	
His[14]	5.11

Figure S1. 2D [^{1}H-^{1}H] TOCSY and 2D [^{1}H-^{1}H ROESY] spectra of RGDechi1-14 (A, B) and ψRGDechi (C, D) peptides acquired at 298K using a 600 MHz spectrometer.

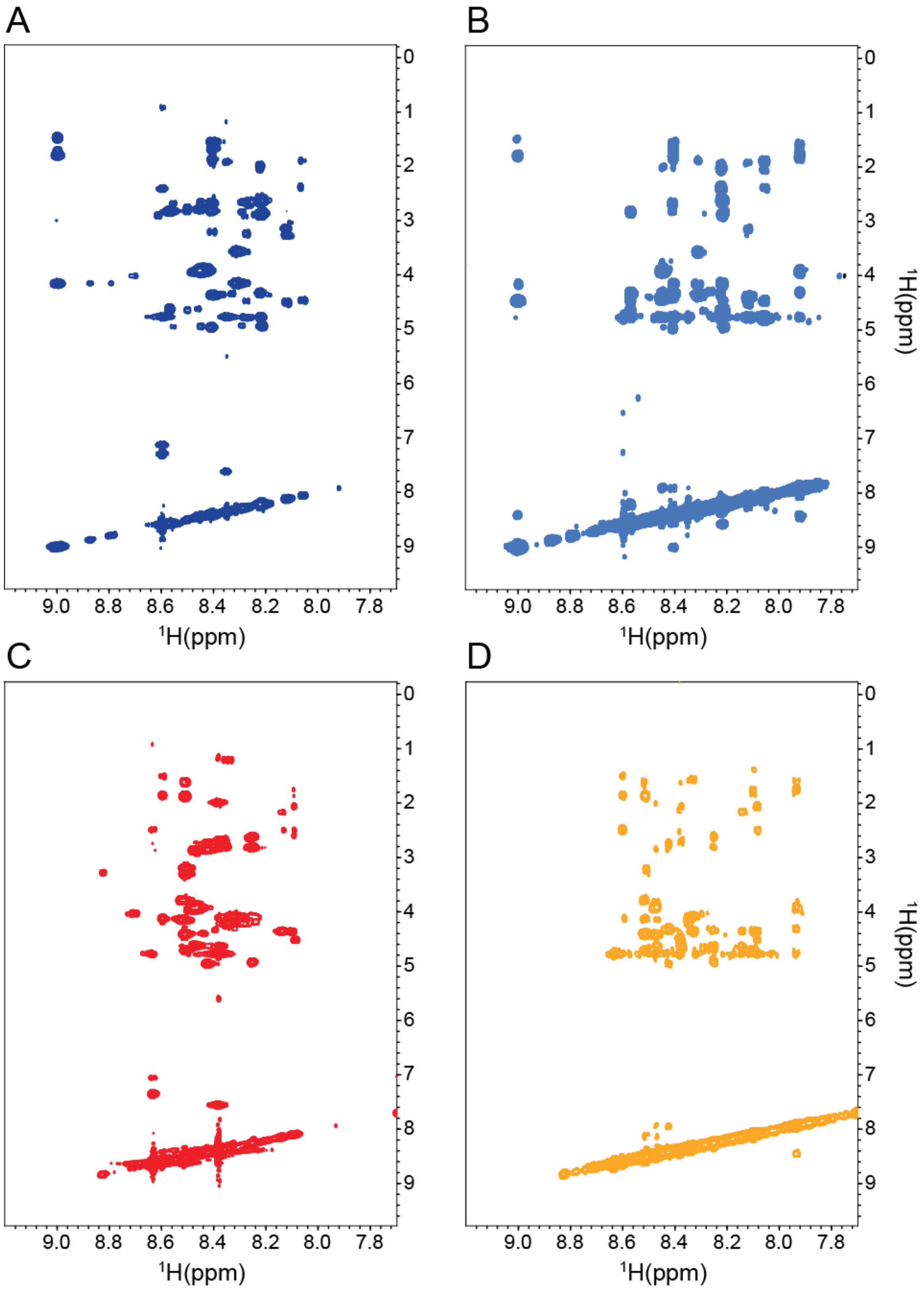

Figure S2. 2D [^{1}H-^{15}N] HSQC spectra of RGDechi1-14 (A) and *ψ*RGDechi (B) peptides acquired at 298K using a 600 MHz spectrometer.

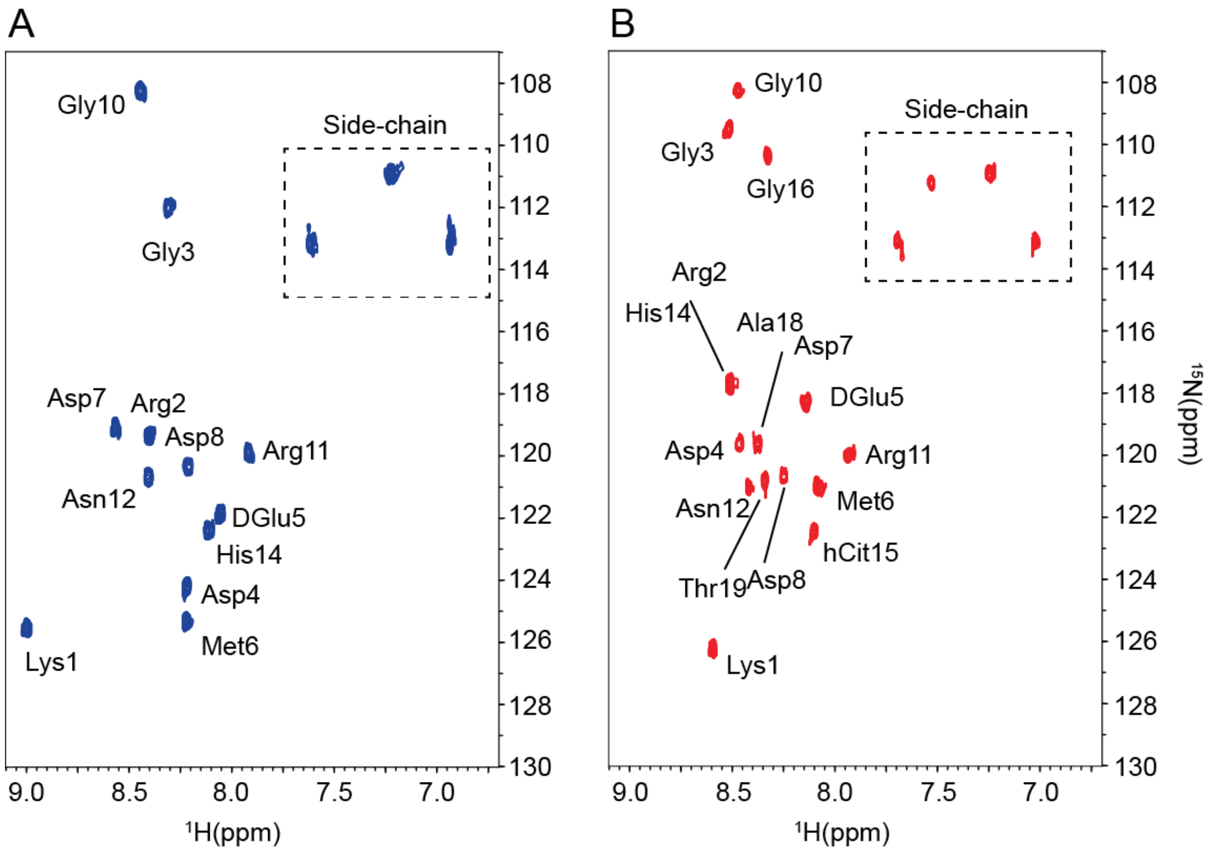

Figure S3. (A) Illustration of the expected NOE/ROE peaks depending on the *cis* and *trans* configuration of the peptidyl- prolyl peptide bond. Arrows indicate protons that are close through the space for each conformation. (Left) In the *cis* configuration of the Xaa-Pro peptide bond an NOE/ROE cross-peak(s) is expected between the Hα of the proline and the Hα of the previous residue. (Right) In the *trans* Xaa-Pro peptide bond configuration NOE/ROE cross-peak(s) are expected between the Hα of the preceding residue and one or both

of the prolyl Hδ2 and Hδ3. (B) A portion of the 2D [^{1}H-^{1}H] ROESY spectra acquired for RGDechi1-14 (Left) and *ψ*RGDechi (Right) displaying Hα-Hδ ROEs from residue pairs D8-P9 and N12-P13 for both peptides and G16-P17 for only *ψ*RGDechi. (C) A portion of the 2D [^{1}H-^{13}C] HSQC spectra acquired on the RGDechi1-14 (Left) and *ψ*RGDechi (Right) at 298 K using a 600 MHz spectrometer. The black boxes indicate the Cβ and Cγ chemical shifts regions for in *cis* and *trans* Xaa-Pro peptide bond conformations.

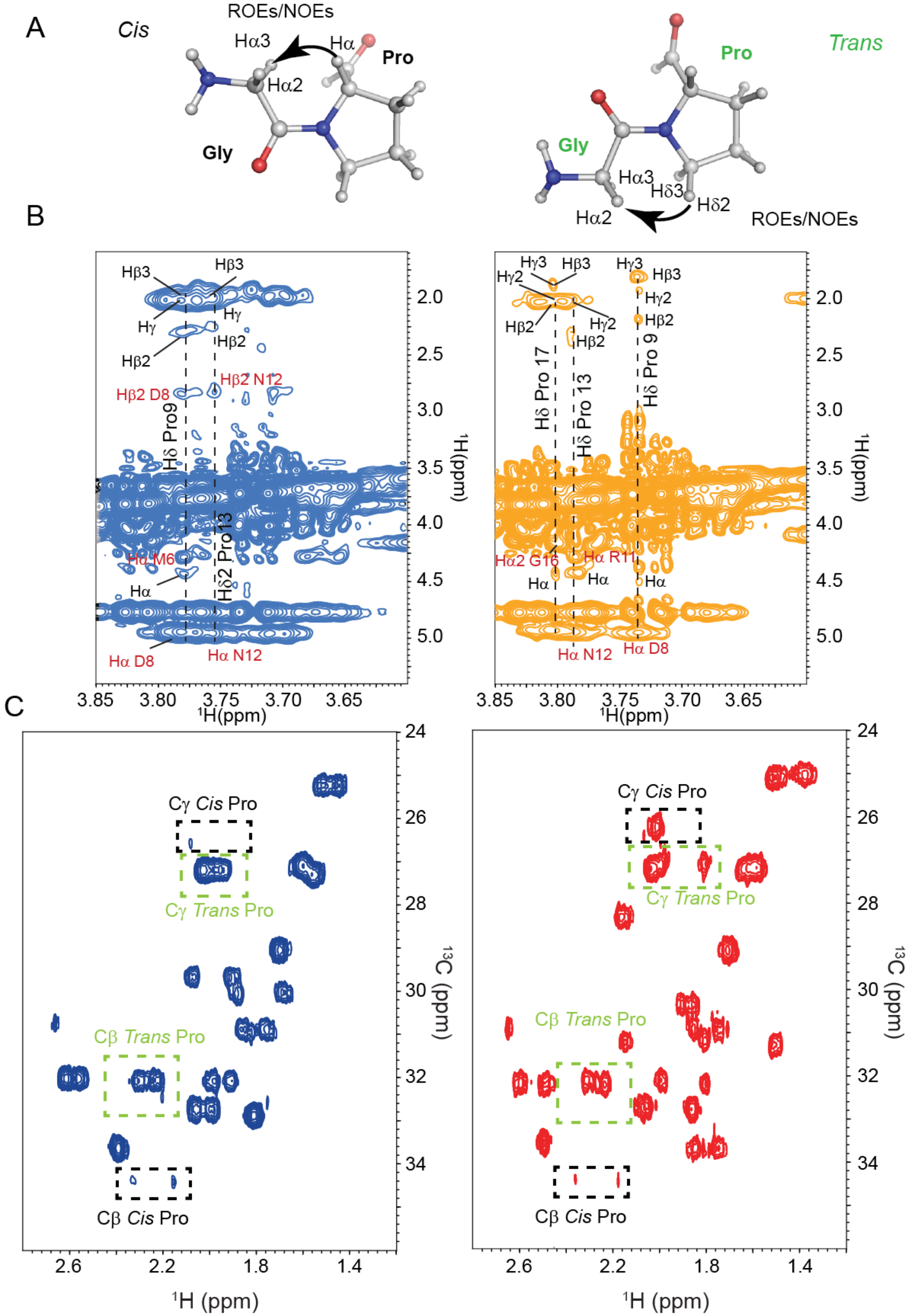

A
Cis
ROEs/NOEs
Hα3
Hα
Pro
Hα2
Gly
Pro
Trans
Gly
Hα3
Hδ3
Hδ2
Hα2
ROEs/NOEs
B
Hβ3
Hβ3
Hγ
Hγ
Hβ2
Hβ2
Hβ2 D8
Hβ2 N12
Hδ Pro9
Hα M6
Hα
Hδ2 Pro13
Hα D8
Hα N12
¹H(ppm)
Hγ3
Hβ3
Hγ3
Hβ3
Hγ2
Hγ2
Hβ2
Hγ2
Hβ2
Hβ2
Hδ Pro 17
Hδ Pro 13
Hδ Pro 9
Hα2 G16
Hα R11
Hα
Hα
Hα
Hα N12
Hα D8
¹H(ppm)
C
Cγ Cis Pro
Cγ Trans Pro
Cβ Trans Pro
Cβ Cis Pro
¹³C (ppm)
¹H (ppm)
Cγ Cis Pro
Cγ Trans Pro
Cβ Trans Pro
Cβ Cis Pro
¹³C (ppm)
¹H (ppm)

Figure S4. Flow-chart of the integrated approach applied to generate the conformational ensemble using a combination of NMR and MD for RGDechi1-14 (A) and *ψ*RGDechi (B) peptides. (C, D) Evaluation by using the experimental ^{1}H, ^{15}N and ^{13}C chemical shifts of each representative structure obtained for RGDechi1-14 and *ψ*RGDechi peptides by analysis of the MD conformational ensemble. The plots report the global CS-RMSD calculated, as reported in the materials and methods, for each model by evaluating the predicted versus observed chemical shifts.

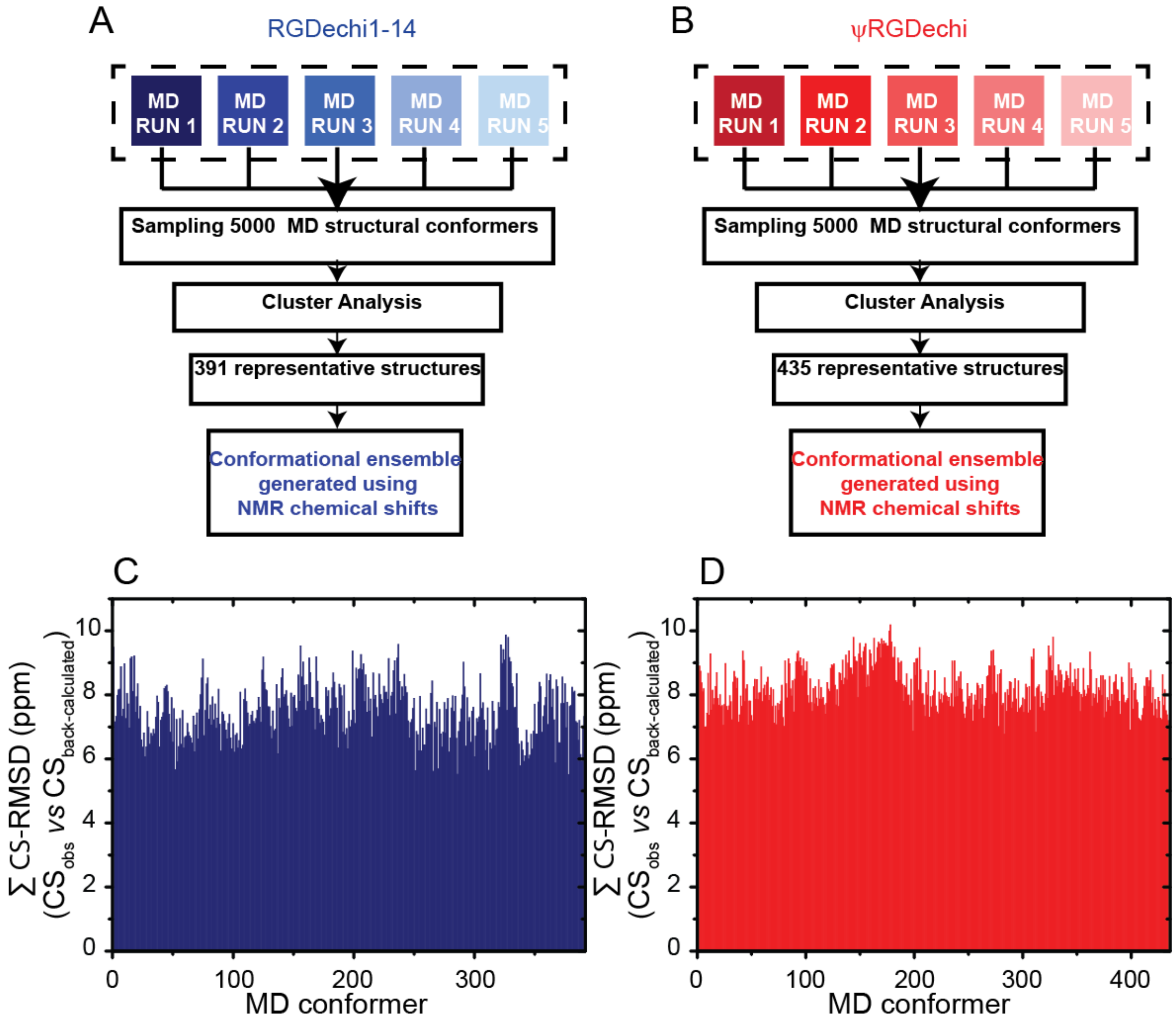

Figure S5. Evaluation of three MD ensembles generated for RGDechi1-14 and ψRGDechi using the experimental ^{1}H, ^{15}N and ^{13}C chemical shifts. The MD ensemble 50x was generated selecting the 50 conformers with the lowest global CS-RMSD; the MD ensemble2 50x was obtained including the 50 conformers with the highest global CS-RMSD; the MD ensemble3 50x was created considering 50 randomly selected conformers. The yellow and blue dash lines indicate the lowest global CS-RMSD values obtained by fitting each MD model for RGDechi1-14 and ψRGDechi, respectively.

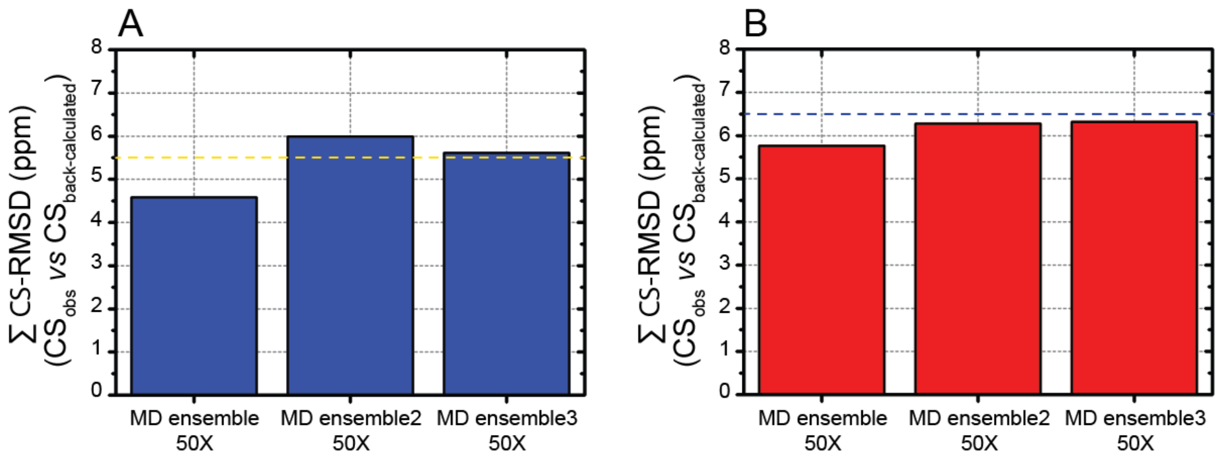

Figure S6. Correspondence between experimental and MD ensemble 50X derived $^{3}J_{HN,H\alpha}$ coupling constants for RGDechi1-14 (A) and ψRGDechi (B).

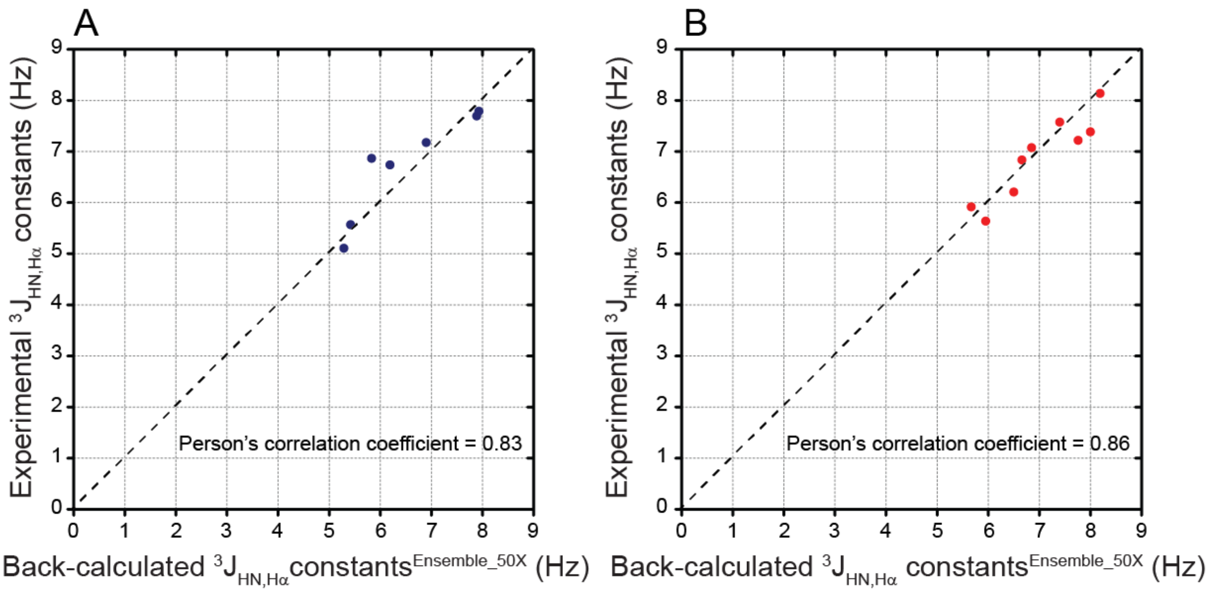

Figure S7. Back-calculated versus experimental proton (A), Cα/Cβ (B) and nitrogen (C) chemical shifts. The predicted shifts were obtained, as reported in the material and methods, using the *ψ*RGDechi-bound conformation reported by Comegna *et al.*. (D) Cumulative ^{1}H,^{13}C and ^{15}N chemical shift differences between predicted and observed shifts plotted as a function of residue number.

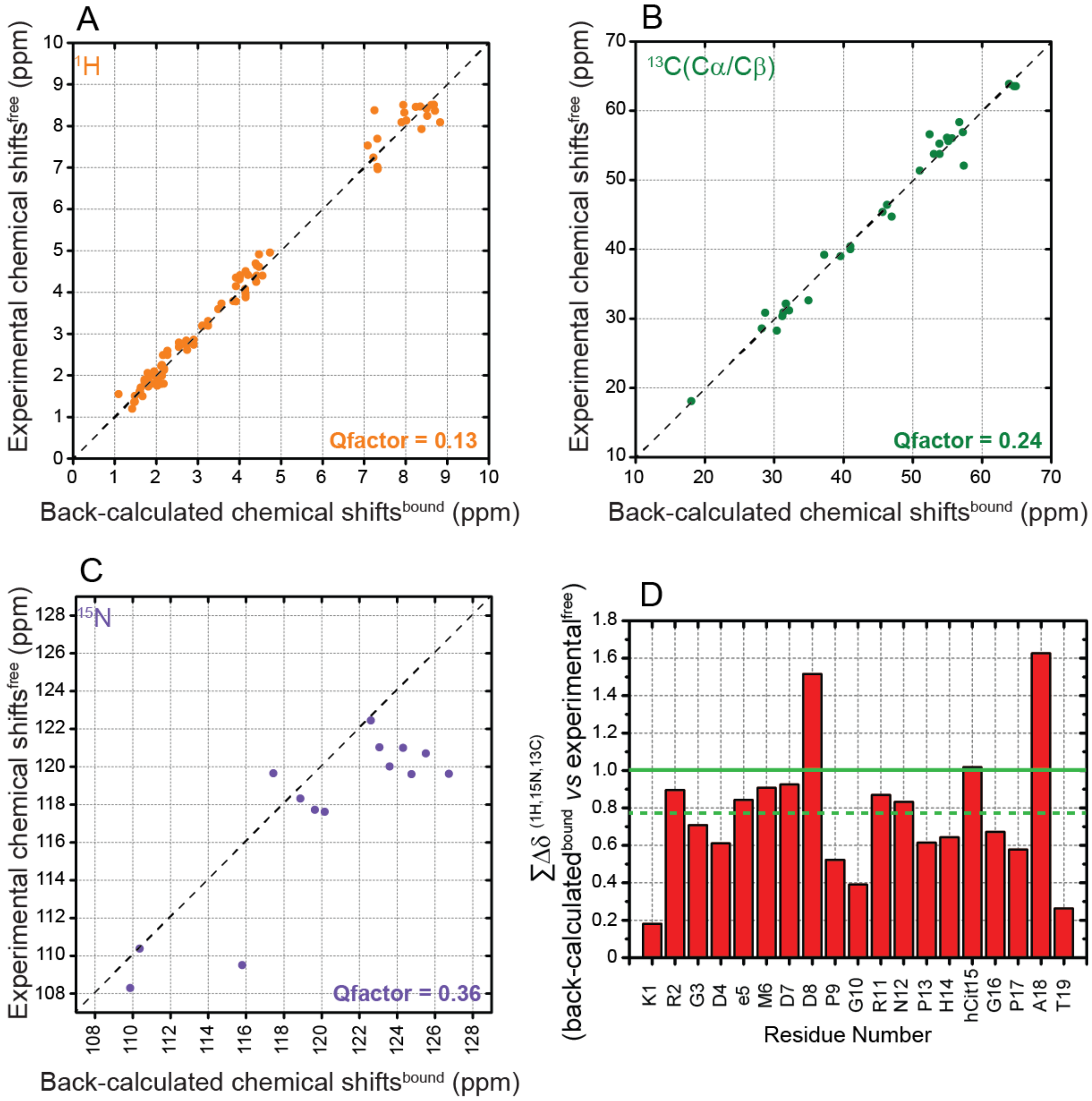

Supplement: Supplementary file 1 [file ijms-23-11039-s001.zip › ijms-1877875-supplementary.pdf]
